# Optimization of the Factors Affecting the Absorption of Vardenafil from Oral Disintegrating Tablets: A Clinical Pharmacokinetic Investigation

**DOI:** 10.3390/pharmaceutics11010011

**Published:** 2019-01-01

**Authors:** Hytham A. Al-Gethmy, Usama A. Fahmy, Nabil A. Alhakamy, Osama A. A. Ahmed, Khalid M. El-Say

**Affiliations:** 1 Department of Pharmaceutics, Faculty of Pharmacy, King Abdulaziz University, Jeddah 21589, Saudi Arabia; hythamalgethmy@hotmail.com (H.A.A.-G.); usamafahmy@hotmail.com (U.A.F.); nalhakamy@kau.edu.sa (N.A.A.); osama712000@gmail.com (O.A.A.A.); 2 Department of Pharmaceutics and Industrial Pharmacy, Faculty of Pharmacy, Minia University, Minia 61519, Egypt; 3 Department of Pharmaceutics and Industrial Pharmacy, Faculty of Pharmacy, Al-Azhar University, Cairo 11651, Egypt

**Keywords:** bioavailability, Box–Behnken design, β-cyclodextrin, erectile dysfunction, taste masking, vardenafil

## Abstract

Because of poor solubility and considerable metabolism, vardenafil (VRD) bioavailability is 15%. To overcome this obstacle, this study aimed to increase the solubility, hasten the onset of action, and mask the unpleasant taste of VRD utilizing β-cyclodextrin (β-CD) and formulation of the inclusion complex as oral disintegrating tablets (ODTs). The solubility of the obtained complexes in various ratios has been studied. A Box–Behnken design (BBD) was utilized to investigate the influence of excipients on the quality of ODTs. The solubility of VRD was improved at 1:2 drug:β-CD ratio. The formulated VRD-ODTs exhibited satisfying results regarding the hardness and disintegration time. In addition, in vivo taste masking and disintegration time showed improved results, after placing the tablets in the oral cavity of the healthy volunteers. When compared with the marketed tablets, the pharmacokinetic parameters for the optimized VRD-ODTs exhibited a significant improvement with *p* < 0.05 in the maximum plasma concentration and reduction in the time needed to reach this concentration. Finally, the optimized VRD-ODTs exhibited increased oral absorption of VRD and subsequent decrease in the time of onset of clinical effect and masking the unpleasant taste.

## 1. Introduction

Vardenafil (VRD) is a potent phosphodiesterase (type V) inhibitor, used for the treatment of the erectile dysfunction disease [1]. Its mechanism of action depends on inhibition of the degradation of cyclic GMP (cGMP) in the smooth muscle tissues located on the internal surface of the blood vessels that supply the corpus cavernosum of the penis. This accumulation of cGMP in the corpus cavernosum leads to the release of nitric oxide that causes dilation of the blood vessels then, the erection occurs successfully [2]. Furthermore, VRD has been utilized in patients with pulmonary hypertension due to the presence of PDE5 in the smooth muscle of the arterial wall within the lungs [1]. According to the biopharmaceutical classification system (BCS), VRD was classified as a class II (high permeability/low solubility) drug [3] that suffers from drawbacks of low bioavailability (15%) and bitter taste. It is also subjected to extensive first-pass metabolism which is one of the reasons for low oral bioavailability. VRD is mainly metabolized by cytochrome P450 [4].

Oral drug delivery is the favored delivery system for numerous drugs. In comparison to different delivery systems, the oral drug delivery has benefits towards of administration and bioavailability [5]. However, due to the difficulties of swallowing facing pediatric, geriatric, and mentally retarded patients, scientists developed oral disintegrating tablets (ODTs) as a convenient mode of administration [6]. Therefore, ODTs improved patient compliance and convenience [7] beside enhanced the absorption and bioavailability compared to conventional tablets [8].

Most active ingredients have unacceptable taste; hence, taste masking has an important role in the formulation of ODTs. The unacceptable taste of the active ingredients can be eliminated by several methods, e.g., the addition of sweeteners and flavors, blending with cyclodextrins (CDs), and encapsulating the unpleasant drug into microparticles [9]. Unacceptable taste is one of the significant drawbacks of orally administered drugs and is encountered with numerous medications. Administration of medications by oral route with pleasant taste is a critical issue for health providers and in the commercial success of ODTs [10]. Therefore, unacceptable tasting drugs often affect the compliance of patients [11]. Masking the unpleasant drug taste could be achieved by several techniques. From these techniques, inclusion complex with CD, ion exchange resins, polymers, and microencapsulation [12,13,14,15,16]. CD complexation ability has been broadly used in pharmaceutics for taste-masking of bitter taste [17,18,19] and improving solubility, stability, and bioavailability of the drug [20,21]. β-cyclodextrin (β-CD) is broadly utilized as a taste covering agent of unacceptable taste drugs due to its solubility (it has the least solubility in comparison to other kinds of CDs), its sweet taste, and its good safety profile [22,23]. The masking of unpleasant taste by CD was superior in β-CD in comparison with gamma and alpha CDs stable complex [15,19]. However, the use of β-CD as a taste-masking agent is broadly reported [24,25,26].

Therefore, the aim of this study was the investigation of the factors affecting the oral absorption of VRD from taste-masked VRD-ODTs. In addition, the pharmacokinetic parameters of the optimized VRD-ODTs were studied on human volunteers.

## 2. Materials and Methods

### 2.1. Materials

Vardenafil (VRD) was purchased from Jinlan Pharm-Drugs Technology Co., Ltd. (Hangzhou, China); β-Cyclodextrin (β-CD) was kindly gifted from Nihon Shokuhin Kako Co., Ltd., (Tokyo, Japan); Crospovidone was from BASF (Ludwigshafen, Germany). Lactose monohydrate (Spectrum, New Brunswick, NJ, USA), microcrystalline cellulose (Avicel PH 101, Sigma-Aldrich, St. Louis, MI, USA), and sodium starch glycolate (Explotab) were kindly gifted from Jamjoom Pharmaceuticals Co. (Jeddah, Saudi Arabia); D-mannitol (Sigma-Aldrich, St. Louis, MI, USA), magnesium stearate and talc from SAJA Pharmaceutical Co. Ltd. (Jeddah, Saudi Arabia), and methanol was purchased from Sigma-Aldrich (St. Louis, MI, USA).

### 2.2. Pre-Formulation Studies

#### 2.2.1. Preparation of VRD-β-CDs Inclusion Complexes

The inclusion complexes of VRD with β-CD at different molar ratios 1:1, 1:1.5, and 1:2 were formed by the kneading method as reported previously [27,28,29,30]. The calculated amounts of VRD and the polymer were triturated with a small volume of methanol to prepare a homogenous slurry, then kneaded for 45 min and dried for 24 h at room temperature. The dried mass was pulverized and passed through mesh No. 200 and stored at 4 °C until further utilization.

#### 2.2.2. Solubility Study

The effect of inclusion complexes on the solubility of VRD was evaluated according to the method reported by Higuchi and Connors [31]. Briefly, an excess of raw VRD and VRD-β-CD were added to vials containing distilled water then placed in a shaking water bath at 25 ± 0.5 °C. Samples were analyzed for VRD content at 230 nm until equilibrium is attained.

### 2.3. Formulation of ODTs

#### 2.3.1. Application of Box Behnken Experimental Design

A three-level three-factor BBD was utilized. These factors are the percentage of a bulking agent (mannitol) as X_1_, the percentage of superdisintegrant (sodium starch glycolate; Explotab) as X_2_, and the percentage of binding agent (microcrystalline cellulose; Avicel) as X_3_. The ODTs hardness (Y_1_), and the disintegration time (Y_2_) were the evaluated responses. Construction and estimation of the statistical design were achieved with Statgraphics Centurion XV version 15.2.05 software (2005), StatPoint Technologies Inc., (Warrenton, VA, USA). The experimental factors and their levels were determined in preliminary studies and the responses with their desirable goals were represented in Table 1. To produce formulations displaying maximum hardness with minimum disintegration time, 15 experimental formulations were suggested by BBD (Table 2).

#### 2.3.2. Preparation of ODTs

Direct compression method was utilized for the preparation of the suggested VRD-ODTs as displayed in Table 2. The tablet blend was compressed using 9 mm flat punches with compression force of 10 KN into 200 mg tablets using a tablet press (Erweka, GmbH, Heusenstamm, Germany).

### 2.4. Evaluation of the Prepared VRD-ODTs

Evaluation of VRD-ODTs was performed on the tablets of all batches considering the visual inspection, weight and content uniformity, thickness, hardness and friability according to the Pharmacopeial requirements.

### 2.5. In Vitro Disintegration of VRD-ODTs

VRD-ODTs (6 tablets/batch) were placed in the baskets of USP disintegration apparatus (Pharmatest, PT-DT7, Hainburg, Germany). The apparatus run utilizing distilled water as the immersion fluid at 37 ± 0.5 °C. The tablets were observed, and the time taken for complete disintegration of all tablets was determined.

### 2.6. In Vitro Dissolution of VRD-ODTs

USP dissolution apparatus II (paddle method) of Erweka GmbH, (Heusenstamm, Germany) was used in the dissolution of VRD from the ODTs. The study was performed with 900 mL distilled water at 50 rpm and equilibrated at 37 ± 0.5°. Samples of 5 mL were withdrawn at the predetermined time intervals 5, 10, 15, 20, 30, 45, and 60 min and replaced with a fresh preheated medium for each time point, then analyzed by UV spectrophotometer at 230 nm. The experiment was performed three times for each formula and the mean values of the cumulative % release of VRD after 60 min were determined.

### 2.7. VRD-ODTs Formulation Data Analysis by BBD

Hardness (Y_1_) and disintegration time (Y_2_) were analyzed using the experimental design software. Significance of the analysis was set for any factor at *p* < 0.05. The optimized VRD-ODT formulation suggested was prepared and checked for the hardness and disintegration time results. The observed values were compared with the predicted ones and the residuals were calculated.

### 2.8. In Vivo Evaluation of the Optimized VRD-ODTs on Human Volunteers

#### 2.8.1. In Vivo Taste Masking and Disintegration Time Evaluation

A single-blind study was intended for disintegration time and the taste masking tests in the buccal cavity of six healthy human volunteers. The study was performed and approved at the Egyptian Research and Development Company (ERDC), Cairo, Egypt on 30 August 2017 with Ethical Approval Code (Verd-p 0566/449). The human subjects were asked to rate the bitter taste of the optimized formula utilizing a scale of 0–3. When the score ≤ 1, the taste was acceptable while if the score >1, indicates the tablet is bitter and not acceptable [14]. Also, the disintegration time of the tablet in the oral cavity was recorded.

#### 2.8.2. Pharmacokinetic Parameters Evaluation

An open-label, single dose, randomized, one-period, parallel design comprising fourteen days of screening preceding 24 h study periods was used. The participants were administered a buccal 10-mg dose of VRD from the optimized formulation tablet (test). While, the marketed tablets (reference) were administered the same dose orally with water. The study was carried out at the Egyptian Research and Development Company (ERDC), Cairo, Egypt. ERDC Research Ethical Committee had formally approved the study design protocol on 30 August 2017 with Ethical Approval Code (Verd-p 0566/449). The study was accomplished in agreement with the Declaration of Helsinki and the International Conference on “Harmonization of Good Clinical Practices”.

##### Population and Sampling

Healthy male volunteers (25–43 years of age) at the time of screening were selected for the study. The selected subjects signed written informed consent, were willing to participate in this clinical trial, and to comply with the study requirements. Complete medical history, laboratory analysis, and physical examination were performed for the selected candidates to ensure their eligibility for participation. Subjects were divided into two groups (6 each). The first group was administered the optimized formulation while the second one was given the marketed VRD tablets. Blood samples (5 mL) were collected at 0, 0.16, 0.25, 0.5, 0.75, 1, 1.25, 1.5, 2, 2.5, 3, 4, 6, 8, 12, and 24 h in heparinized tubes. The tubes were centrifuged at 3500 rpm for 10 min (Centurion, West Sussex, UK) and the separated serum was stored at −80 °C until analysis.

##### Chromatographic Conditions

VRD detection in human plasma was conducted using a high-performance liquid chromatography coupled with MS/MS method (HPLC-MS/MS) that was developed at the ERDC according to the reported method with slight modification [32]. Validation of the method was based on the FDA Bio-analytical Method Validation Guidelines 2003. Assay linearity was verified for VRD at a concentration range of 3–350 ng/mL with regression coefficients (R^2^) of 0.996 and 0.994 for VRD. The lower limits of quantification were 3 ng/mL for VRD. The HPLC-MS/MS system consisted of HPLC, Agilent series 1200 (Agilent Technologies Deutschland GmbH, Waldbronn, Germany), used with a Triple Quad G1311A quaternary pump equipped with 6400 Series Triple Quadrupole LC/MS detector and mass hunter software. Chromatography was performed (75% acetonitrile: 25% buffer (ammonium formate 20 mM + 0.2% (*v*/*v*) formic acid in water) as mobile phase at a flow rate of 0.35 ml/min and a reversed phase column Intersil ODS-3 (4.6 mm × 50 cm, dp 5µm) at 25 °C. Sildenafil was selected as an internal standard (IS).

##### Pharmacokinetic Analysis

A noncompartmental analysis of the pharmacokinetic parameters was achieved by unpaired *t*-test (two-tailed) using Kinetica™ software (Version 4; Thermo Fisher Scientific, Waltham, MA, USA, 2005). Any significant difference in drug plasma concentration between the two groups was assessed with two-way ANOVA followed by Sidak’s multicomparison test using GraphPad Prism 6 (GraphPad Software, Inc., San Diego, CA, USA, 2012). Results were considered significant at *p* < 0.05. The peak plasma concentration achieved by the drug (C_max_), the time after administration of a drug when the maximum plasma concentration is reached (t_max_), the area under curve (AUC), elimination rate constant (K_el_) and mean drug residence time (MRT) was calculated to allow the relative bioavailability [(AUCformulation/AUCtablets) × 100] to be determined.

## 3. Results and Discussion

VRD-ODTs were developed to deliver the drug rapidly. A pre-formulation study involving complexation of VRD with β-CD was carried out. A direct compression method was used for the formulation of 15 formulae of VRD-ODTs according to BBD. All the prepared formulations were evaluated for weight uniformity, thickness, friability, hardness, content uniformity, and in vitro disintegration as well as in vitro dissolution. The results of all experiments were used to correlate the independent variables that constitute the combination of excipients of tablets with the dependent variables that represent the quality parameters of the ODTs. BBD utilized these relations to statistically optimize the process to produce VRD-ODTs with maximum hardness and minimum disintegration time. Finally, the obtained optimized formulation was evaluated in vivo on healthy human volunteers compared with the marketed Levitra tablets. The details of the results and their discussion are given in the following sections.

### 3.1. Saturation Solubility Studies of the Prepared Complexes

**Figure 1:**
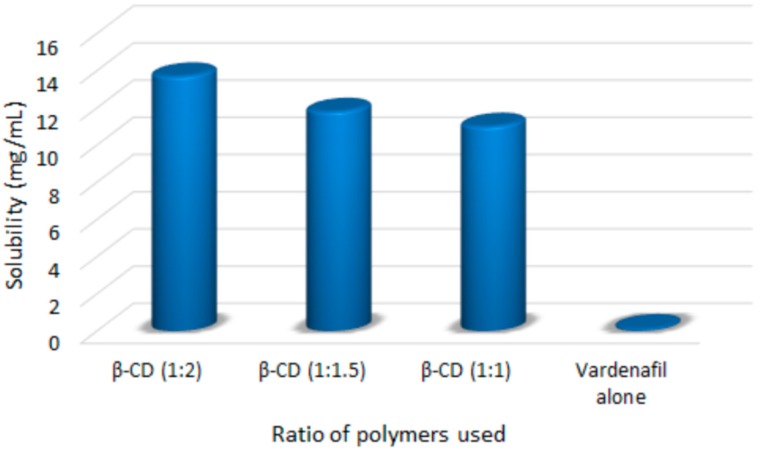
The solubility of VRD in complexes with the β-CD polymer in different ratios.

The data represented in Figure 1 revealed that the solubility of raw VRD was 0.13 mg/mL. While the solubility of VRD in solid dispersion using β-CD in different molar ratios 1:1, 1:1.5, and 1:2 was increased to reach 13.7 mg/mL for VRD-β-CD 1:2 molar ratio. This finding confirms the formation of a complex with β-CD and improves VRD solubility which in a good agreement with the previously reported results of carvedilol [33], piroxicam [34], ketoconazole [35], gliclazide [36], zafirlukast [37], and aripiprazole [38].

### 3.2. Development of VRD–ODTs

Development of the formulation in the present study was mainly based on three factors namely mannitol percentage as a diluent, Explotab percentage as a superdisintegrant, and Avicel percentage as a binder. Various ratios of each component combinations were used to get oral disintegrating tablets with good quality attributes. All formulations were suggested by BBD as described in Table 1.

### 3.3. Evaluation of VRD-ODTs

All batches of VRD-ODTs were evaluated and results are shown in Table 3. The tablet weight variation of the prepared batches was less than 2%, in accordance with USP requirements. The tablets formulations met the European Pharmacopeia limits for disintegration of oral disintegration tablets (<3 min). Friability, hardness and thickness of the prepared tablets were in the acceptable limits as indicated in Table 3.

In addition, drug content was in the range of 95.43–101.7%. To avoid delaying the disintegration of ODTs, the hardness usually planned to be lower than the conventional tablets. The hardness is an essential factor which affects the disintegration and dissolution times that influence bioavailability [39]. Some challenges encountered in the formulation and production of ODTs such as the disintegration time and mechanical strength. The ideal ODTs should have concise disintegration time that is usually about one min or less which require low mechanical strength. The disintegration time is directly proportional with the mechanical strength of the tablets. Therefore, it is essential to have a good compromise between mechanical strength and disintegration time [9].

### 3.4. In Vitro Dissolution Studies

**Figure 2:**
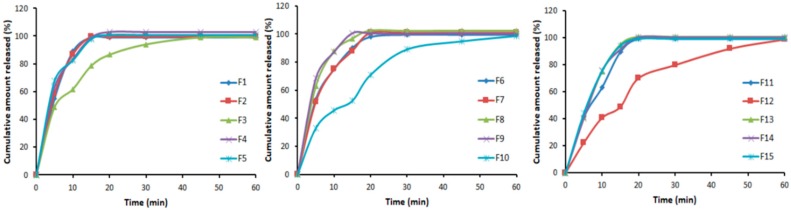
In vitro dissolution profile of formulations (F1–F15).

Figure 2 displayed the release profiles of the 15 formulations proposed by BBD. Most of the formulations released all VRD content within 20 min of the study period and all of them showed more than 98.56% within 60 min.

### 3.5. Quantitative Estimation of the Factors Affecting VRD-ODTs

Table 4 shows the estimate effect of each factor, F-ratio and p-values for Y_1_ and Y_2_ from two-way analysis of variance (ANOVA). From the obtained analysis, Explotab percentage (X_2_) had no significant effect on the hardness of tablets (Y_1_) but had a significant antagonistic effect on the disintegration time of the tablets (Y_2_) with a P-value of 0.0001. Also, it was found that mannitol percentage (X_1_) and Avicel percentage (X_3_) had significant synergistic effects on the hardness (Y_1_) with *p*-values of 0.0009 and 0.0001, respectively. In addition, the interaction term X_1_X_2_ showed a significant synergistic effect on the hardness with a *p*-value of 0.0035. The R-Squared statistic indicates that the model as fitted explains 97.647% and 82.457% of the variability in hardness and disintegration time, respectively. 

#### 3.5.1. Mathematical Modeling of the Data

Mathematical modelling for Y_1_ and Y_2_ of VRD-ODTs were generated after analysis of the data using the Statgraphics^®^ software (Equations (1) and (2)).

Hardness (Y_1_) = 34.332 − 1.360 X_1_ − 1.986 X_2_ − 0.244 X_3_ + 0.011 X_1_^2^ + 0.073 X_1_X_2_ + 0.006 X_1_X_3_ − 0.002 X_2_^2^ − 0.028 X_2_X_3_ + 0.0119 X_3_^2^ (1)

In vitro disintegration time (Y_2_) = 311.603 − 21.762 X_1_ + 32.988 X_2_ + 0.674 X_3_ + 0.248 X_1_^2^ − 0.106 X_1_X_2_ + 0.342 X_1_X_3_ − 2.001 X_2_^2^ − 0.333 X_2_X_3_ − 0.229 X_3_^2^ (2)

**Figure 3:**
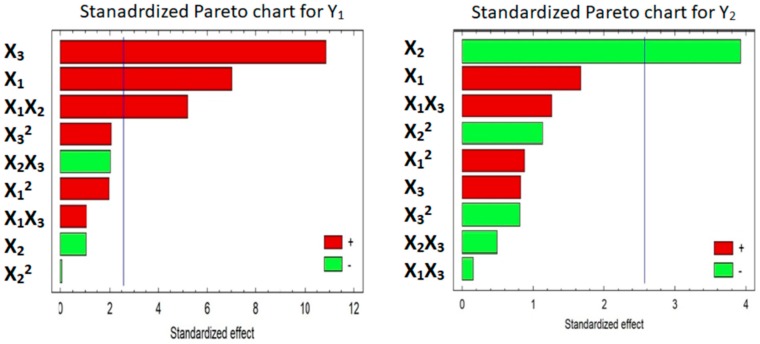
Standardized Pareto charts for Y_1_ and Y_2_. Abbreviations: Hardness (Y_1_), Disintegration Time (Y_2_), Avicel% (X_3_), Explotab% (X_2_), mannitol% (X_1_).

Equations (1) and (2) reflect the quantitative effect of formulation factors; mannitol % (X_1_), Explotab % (X_2_), and Avicel % (X_3_) and their interactions on the responses; the hardness (Y_1_) and the in vitro disintegration time (Y_2_). Figure 3, 2D Pareto charts, showed the effect of X_1_–X_3_ and their interactions on Y_1_ and Y_2_.

#### 3.5.2. Effect of the Factors on Y_1_ and Y_2_

**Figure 4:**
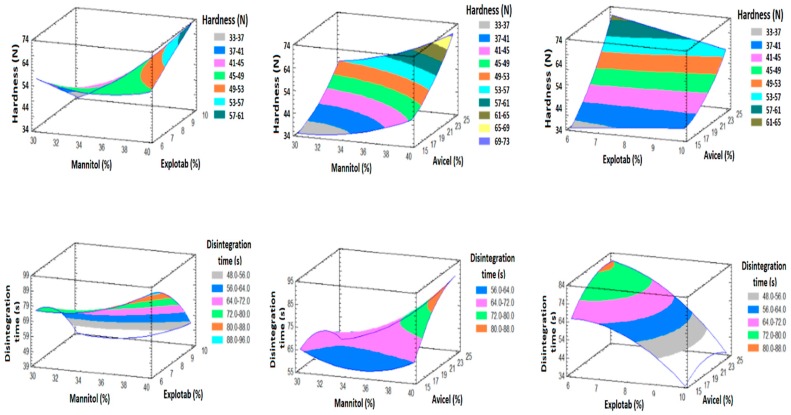
Response surface plots (3D) showing the effects of X_1_, X_2_ and X_3_ on Y_1_ and Y_2_.

Figure 3 displayed that X_1_ and X_3_ have significant synergistic effects on Y_1_, and the interaction between X_1_ and X_2_ have also a significant synergistic effect on Y_1_. While X_2_ has no significant effect on Y_1_. Figure 4, 3D response surface plots, confirmed the understanding of the influence of each factor on Y_1_, Similar findings were reported in the literature for the effect of excipients on the prepared tablet properties [40,41,42,43,44,45,46].

Also, Figure 3 displayed that X_2_ has a significant antagonistic effect on Y_2_. This finding means that increasing X_2_ level will lead to reduction in the disintegration time. Other studies have investigated the effect of Explotab on tablet disintegration times [43,47,48]. Both X_1_ and X_3_ have no significant effect on Y_2_. Also, 3D response surface plots (Figure 4) were graphically established utilizing the software.

### 3.6. Prediction of the Optimized Formulation

The optimized formulation of VRD-ODTs was predicted upon the data analysis with desirability function equal to 0.883603 over the indicated region. The optimum combination of factors was 38.52% of Mannitol concentration, 9.99% of Explotab concentration, and 25% of Avicel concentration. The observed, predicted and residual values for Y_1_ were 68.45 N, 65.90 N and 2.55 N, respectively while, for Y_2_ were 51.77 s, 49.78 s and 1.99 s, respectively. This finding supports the mathematical experimental design in maximizing Y_1_ and minimizing Y_2_ that fulfills the Pharmacopoeial requirements via the direct compression method.

### 3.7. In Vivo Evaluation of the Optimized VRD-ODTs on Human Volunteers

#### 3.7.1. In Vivo Taste Masking and Disintegration Time

The results of the in vivo taste masking test were listed in Table 5. The scores of the six volunteers were equal or less the one which indicate the formulation has an acceptable taste masking effect. The mean result of the in vivo disintegration time was 62.33 s which is acceptable according to European Pharmacopeia (<3 min).

#### 3.7.2. Pharmacokinetic Parameters Evaluation

**Figure 5:**
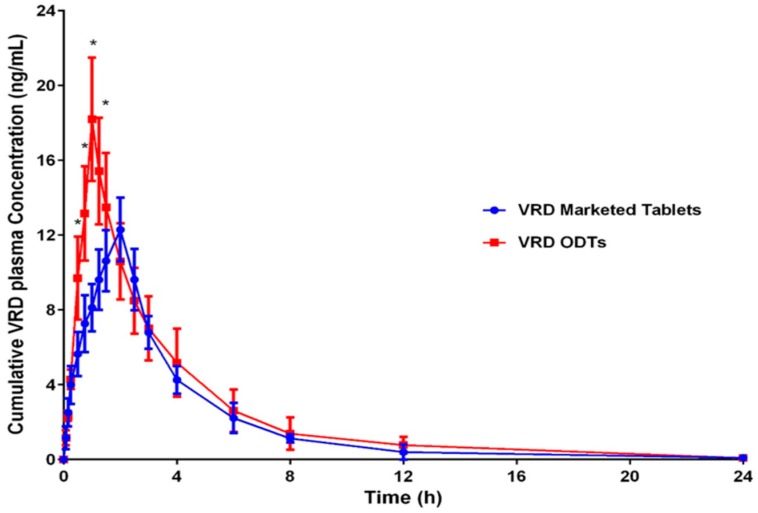
The mean plasma concentration-time profiles of VRD after oral administration of a single oral dose (10 mg) of the marketed Levitra tablet and optimized VRD-ODTs (n = 6).

The VRD plasma concentration time profiles from the optimized formulation of VRD-ODT and the marketed Levitra tablet (Bayer AG, Leverkusen, Germany) are represented in Figure 5. The values of C_max_, t_max_ and AUC_(0–24)_, t_1/2_, K_el_, and MRT for VRD from these formulations, are summarized in Table 6. The results indicated that optimized VRD-ODTs bioavailability (F) compared with the marketed tablet was 125.445%. This data indicated that ODT’s improved the bioavailability of VRD over the marketed tablets. The oral absorption of VRD from ODTs was obviously higher when compared with the marketed tablets which was obvious from the value of C_max_ that increased significantly from 12.29 ng/mL for the marketed tablet to 18.19 ng/mL (for the optimized VRD-ODTs.

In addition, the t_max_ of optimized VRD-ODTs shortened to 1 h when compared with t_max_ of 2 h for the marketed tablets which indicated that the onset of action of VRD from optimized ODTs was accelerated in comparison with the marketed tablets. The analysis of variance showed that there were significant differences among the samples (*p* < 0.05) taken at 0.5, 0.75, 1, and 1.25 h from the two groups of volunteers indicating the significant improvement achieved by the ODTs. Complexation of VRD with β-CD followed by its formulation as taste masked ODTs enhance both the solubility and dissolution rate that reflects on the availability of VRD ready for absorption [49]. Accordingly, optimized VRD-ODTs is a promising approach for improved VRD bioavailability and absorption rate.

## 4. Conclusions

The present study proves that the inclusion complex of VRD with β-CD increased its aqueous solubility in a 1:2 molar ratio and masked its bitter taste. The optimized formulation compromises between the hardness and the disintegration time. The studied tablets’ excipients showed the varied impact to fulfill all the required characteristics of ODTs. However, incorporation of an optimized concentrations, via using the design of the experiment, of these excipients achieved the best balance and therefore produced VRD-ODTs with superior characteristics. The developed tablet offers a taste-masked, satisfactory hardness, and short disintegration time for the rapid release of the drug. The pharmacokinetic data point out to the improved bioavailability of VRD over the marketed tablets. In addition, in vivo data found that the oral absorption of VRD from ODTs was obviously higher than that from the marketed tablets. Moreover, the t_max_ was shortened to 1 h in comparison with 2 h for the marketed tablets which indicated the rapidity of onset of action of VRD and hence improved patient efficacy and satisfaction.

## Figures and Tables

**Table 1:** Factors and their levels with the desirable goals of the responses.

Factors	−1	0	+1	Response	Goal	Desirability
Mannitol (%)	30	35	40	Hardness (N)	Maximize	70 N
Explotab (%)	6	8	10	Disintegration time (s)	Minimize	30 s
Avicel (%)	15	20	25			

**Table 2:** Composition of vardenafil oral disintegrating tablets formulations based on Box–Behnken design.

Formula #	Drug Complex *	Mannitol	Explotab	Avicel	Crospovidone	Lactose	Magnesium Stearate	Talc
	(mg)							
**F1**	30	70	20	30	10	36	2	2
**F2**	30	70	20	50	10	16	2	2
**F3**	30	60	12	40	10	44	2	2
**F4**	30	70	12	30	10	44	2	2
**F5**	30	60	16	50	10	30	2	2
**F6**	30	80	16	30	10	30	2	2
**F7**	30	70	12	50	10	24	2	2
**F8**	30	60	20	40	10	36	2	2
**F9**	30	80	20	40	10	16	2	2
**F10**	30	80	16	50	10	10	2	2
**F11**	30	60	16	30	10	50	2	2
**F12**	30	80	12	40	10	24	2	2
**F13**	30	70	16	40	10	30	2	2
**F14**	30	70	16	40	10	30	2	2
**F15**	30	70	16	40	10	30	2	2

* Equivalent to 5 mg VRD.

**Table 3:** Characteristics of VRD-ODTs, data expressed as mean ± SD (n = 10).

Formula #	Weight (mg)	Thickness (mm)	Hardness (N)	Friability (%)	Drug Content (%)	Disintegration Time (s)
**F1**	203.0 ± 3.06	2.64 ± 0.015	36.482 ± 0.26	0.635	97.77 ± 0.04	38.32 ± 5.77
**F2**	201.6 ± 0.57	2.62 ± 0.035	54.723 ± 0.21	0.496	101.07 ± 0.08	48.67 ± 7.64
**F3**	203.3 ± 1.15	2.60 ± 0.003	46.485 ± 0.15	0.147	100.98± 0.01	88.33 ± 10.4
**F4**	204.3 ± 0.57	2.60 ± 0.001	33.736 ± 0.10	0.645	99.37 ± 0.04	52.67 ± 2.51
**F5**	202.3 ± 1.53	2.69 ± 0.006	52.075 ± 0.21	0.494	101.7 ± 0.04	46.67 ± 5.77
**F6**	202.6 ± 0.57	2.69 ± 0.006	44.817 ± 0.31	0.493	98.7 ± 0.08	72.67 ± 2.51
**F7**	200.0 ± 1.53	2.60 ± 0.000	63.059 ± 0.12	0.301	96.84 ± 0.05	76.33 ± 3.21
**F8**	201.6 ± 1.15	2.60 ± 0.001	31.088 ± 0.06	0.297	99.86 ± 0.05	36.33 ± 3.21
**F9**	203.0 ± 1.0	2.57 ± 0.025	61.489 ± 0.50	0.197	99.73 ± 0.01	41.43 ± 2.88
**F10**	203.0 ± 1.0	2.59 ± 0.010	65.903 ± 0.06	0.492	100.09 ± 0.04	88.49 ± 7.63
**F11**	199.3 ± 0.57	2.60 ± 0.001	36.776 ± 0.21	0.051	100.45 ± 0.04	65.0 ± 10.0
**F12**	203.0 ± 2.08	2.60 ± 0.001	48.447 ± 0.38	0.977	97.59 ± 0.01	97.67 ± 8.73
**F13**	201.0 ± 1.53	2.60 ± 0.341	42.170 ± 0.16	0.299	95.43 ± 0.04	66.43 ± 5.13
**F14**	202.0 ± 1.53	2.60 ± 0.001	44.524 ± 0.11	0.098	98.84 ± 0.04	73.33 ± 5.77
**F15**	202.0 ± 1.0	2.61 ± 0.001	45.799 ± 0.10	0.198	100.36 ± 0.08	63.45 ± 2.88

**Table 4:** Analysis of variance and lack of fit parameters of testing the model in portions and estimated effects of factors, *F*-ratios and associated *p*-values for (Y_1_ and Y_2_) responses.

Factor	Hardness (Y_1_)			Disintegration Time (Y_2_)		
	Estimate	*F*-Ratio	*p*-Value	Estimate	*F*-Ratio	*p*-Value
A: Mannitol	1.3825	49.05	0.0009 *	15.9825	2.78	0.1565
B: Explotab	−0.2025	1.05	0.3520	−37.5625	15.34	0.0112 *
C: Avicel	2.14	117.53	0.0001 *	7.875	0.67	0.4490
AA	0.5717	3.87	0.1063	12.4133	0.77	0.4195
AB	1.45	26.98	0.0035 *	−2.12	0.02	0.8819
AC	0.295	1.12	0.3390	17.075	1.58	0.2637
BB	−0.0183	0.00	0.9521	−16.0067	1.29	0.3083
BC	−0.565	4.10	0.0989	−6.655	0.24	0.6445
CC	0.5967	4.22	0.0952	−11.4717	0.66	0.4534
R^2^ (%)	97.647			82.457		
Adjusted R^2^ (%)	93.411			50.879		
**Analysis of Variance**						
*F*-ratio	23.2920			2.6039		
*p*-value	0.0015			0.1522		
**Lack of Fit**						
*F* ratio	2.8493			11.2529		
*p* value	0.2705			0.0827		
R^2^	0.9956			0.9902		

* Significant effect of factors on the investigated response.

**Table 5:** In vivo taste masking and disintegration time of the optimized formula.

Volunteer No.	Disintegration Time (s)	Taste Masking (0–3)
V1	65	0
V2	60	1
V3	62	1
V4	63	0
V5	59	0
V6	65	0
Mean	62.33 ± 2.503	

**Table 6:** Pharmacokinetic parameters ± SD of VRD following the administration of a single oral dose (10 mg) of either the VRD marketed tablet, or the optimized VRD-ODTs.

Pharmacokinetic Parameter	VRD Marketed Tablet	Optimized VRD-ODTs
C_max_ (ng/mL)	12.29383 ± 2.55	18.191 ± 1.95 *
t_max_ (h)	2.0 ± 0.13	1.0 ± 0.21 *
AUC_(0–24)_ (ng·h/mL)	46.68801 ± 4.63	58.81263 ± 5.15
AUC_(24–∞)_ (ng·h/mL)	0.460347 ± 0.34	0.332505 ± 0.17
AUC_(0–∞)_ (ng·h/mL)	47.14836 ± 3.61	59.14514 ± 17.35
AUMC_(0–24)_ ng·hr^2^/mL	184.5833	239.0714
AUMC_(24–end)_ ng·hr^2^/mL	11.04833	7.980117
AUMC_(0–end)_ ng·hr^2^/mL	195.6317	247.0515
K_el_ (h^−1^)	0.209 ± 0.01	0.230573
t_1/2_ (h)	3.317 ± 0.63	3.005555 ± 0.53
MRT (h)	4.149 ± 0.93	4.174615 ± 1.33
CL (mL/h)	3.824974	2.929219
Relative bioavailability (%)	100	125.445

* Significant difference at *p* < 0.05 (unpaired *t* test). VRD, Vardenafil; ODTs, oral disintegrating tablets; AUC, area under the time–concentration curve; C_max_, maximum plasma concentration; K_el_, elimination rate constant; MRT, mean residence time; t_max_, time to reach C_max_.
